# Nano Packaged Tamoxifen and Curcumin; Effective Formulation against Sensitive and Resistant MCF-7 Cells

**Published:** 2018

**Authors:** Samira Hajigholami, Ziba Veisi Malekshahi, Narges Bodaghabadi, Farhod Najafi, Hadi Shirzad, Majid Sadeghizadeh

**Affiliations:** a *Department of Genetics, Faculty of Biological Sciences, Tarbiat Modares University, Tehran, Iran.*; b *Department of Medical Biotechnology, School of Advanced Medical Sciences and Technologies, Tehran University of Medical Sciences, Tehran, Iran. *; c *Department of Resin and Additives, Institutes for Color Sciences and Technology, Tehran, Iran.*; d *Department of Medical Genetics, Faculty of Medical Sciences, TarbiatModares University, Tehran, Iran.*

**Keywords:** Breast Cancer, Curcumin, Tamoxifen, Polymer Nano-carrier

## Abstract

Tamoxifen is routinely used for treatment of Estrogen-positive breast carcinoma. Approximately, 50% of patients with metastatic cancer will develop resistance to Tamoxifen. In this research, Tamoxifen was combined with the anti-cancer compound Curcumin. Diblocknanopolymer was used to package the new formulation of Curcumin and Tamoxifen. Anti-cancer efficacy of the obtained compound was evaluated in Tamoxifen-sensitive (TS). MCF-7, Tamoxifen-resistant (TR) MCF-7 cancer cells and Fibroblast cells. MTT assay was used to evaluate anti-proliferation and toxicity. Flow cytometry and Annexin-V-FLUOS were used to assay anti-proliferation and induction of apoptosis respectively. Our results indicate that the obtained nano-compound is less toxic to normal cells compared to Tamoxifen alone, and has higher anti-proliferation and pro-apoptotic activity on TS-MCF-7 and TR-MCF-7. The nanopolymer reduces the Tamoxifen toxicity in normal cells and counters the developed resistance to the drug in cancer cells.

## Introduction

Breast cancer is the most common type of cancer among women. This cancer comprises 22% of the aggressive cancers and 18% of the all cancers in women (1, 2). Estrogen receptor positive (ER^+^) breast cancer includes 70% of all types of breast cancers and is usually treated with Tamoxifen which is an estrogen receptor antagonist (3). Although Tamoxifen is considered as an effective drug of choice in ER^+^ breast cancer, its long-term usage is accompanied by various side-effects in normal tissues. These side-effects include; higher risk for endometrial and liver cancers (4), risk of venous thromboembolism (5) and damages to the central nervous system and skeleton growth (6, 7). Utilization of lower dosages of the drug reduces these side-effects, however, this will also cause poor anti-tumor outcome and lowers induction of apoptosis in ER^+^ cancer cells. Incorporating a biological compound with lower toxicity than Tamoxifen and same or higher anti-tumor effects will result in safer and more effective treatment of breast cancer. 

Curcumin is a al poly-phenol and hydrophobic anti-cancer compound which is extracted from the roots of Turmeric (*Curcuma longa*) (8, 9). Curcumin can alter the expression of genes involved in the cancer signaling pathway to affect and inhibit cancer initiation, promotion, tumor cell survival and proliferation, angiogenesis, and metastasis stages (9). Curcumin induces apoptosis in cancer cells and increases the population of cells with sub-G0/G1 DNA content. The mechanism of action is overexpression of pro-apoptotic BAX in downstream of P53 pathway (10). 

Reducing the dosage of Tamoxifen significantly reduces the toxicity of the drug. The addition of Curcumin to the reduced dosages of Tamoxifen can restore or even improve the anti-tumor impact of the Tamoxifen and induce apoptosis in cancer cells without affecting normal cell population. 

One of the most significant consequences of long-term usage of Tamoxifen is the development of drug-resistant cancer cells which continuously proliferate even in the presence of high dosage of the drug. Over 50% of patients suffering from metastatic breast cancer will develop *de novo* resistance to the Tamoxifen and eventually all patients will develop Tamoxifen-resistant cells (4, 11, 12). 

Approximately, 40% of patients suffering from breast cancer develop resistance to Tamoxifen after 1-3 years of therapy (3). Curcumin can overcome various mechanisms of Tamoxifen resistance by affecting gene-expression profile of cancer cells, resulting in apoptosis in cancer cell by lower dosages of the drug (3). 

Curcumin is an unstable lipophilic polyphenol and is rapidly metabolized by glucuronidation in the liver and intestines and passes the body through feces (13, 14). 

In this research, we used Diblock (dendrosome) which is a nontoxic nano-carrier to increase bio-availability of Curcumin. Dendrosomes are natural, biodegradable amphipathic nano-carriers and have been previously used by the authors’ research group to deliver genes and Curcumin to cancer cells (15-17). In addition to increase the half-life of Curcumin, the polymer nano-carrier will also provide packaging of Tamoxifen and Curcumin to be co-delivered to cancer cells.

## Experimental

*Materials and Methods*

*Cell Culture*

MCF-7 cells and Fibroblast cells were cultured in high glucose DMEM medium (GIBCO, USA) supplemented with 10% FBS (GIBCO, USA) and 1% penicillin/streptomycin. The culture condition for all cell types were 37^o^C, 5% CO_2 _and 80% humidity. All cells were sub-cultured when reached 80% confluency.

*Preparation of Compounds*

A Total of four compounds were used in this research. Tamoxifen (Sigma-Aldrich, USA), nano-Curcumin (nanoCur) (Curcumin was purchased from Sigma-Aldrich, USA), nano-Tamoxifen (nanoTam) and nano-(Tamoxifen+Curcumin) (nanoTC).powder of Diblock polymer (OM200) was used for preparation of nano-carrier. The composition ratio of nano-compounds were: one part (*w/w*) of the compound and five parts (*w/w*) of the Diblock for nanoTam and nanoCur. NanoTC was developed from one part Tamoxifen, three parts (*w/w*) Curcumin and twenty parts (*w/w*) nano-polymer. The procedure was carried out as described before (16-17).

To simplify, hereafter, only the concentration of Tamoxifen will be mentioned for nanoTam and nanoTC and concentration of Curcumin will be mentioned for nanoCur. The ratio of compound to dendrosome was chosen based on obtaining maximum solubility and avoiding formation of sediments. For NanoTC, the ratio of 3:1 for curcumin:Dendrosome, was the maximum amount of curcumin to dendrosome that did not result in the formation of sedimentation. 

*Establishing Tamoxifen-Resistant MCF-7 cells*

MCF-7 cells were continuously treated with Tamoxifen to establish Tamoxifen-resistant MCF-7 cells. Tamoxifen was added to the culture at the concentration of one unit below IC50. The concentration of Tamoxifen was increased gradually for the duration of four months. A total of 15 passages were carried out to obtain Tamoxifen resistant cells. At this point cells were resistant to two folds of the normal Tamoxifen IC50. The procedure was carried out as described before (23)

*Cell Viability Assay*

MethylthiaozolTetrazolium (MTT) assay was used to assess the cell viability after treatments of cells with each compound. Cells were collected by trypsinization at 70% confluency and seeded into wells of 96-well ELISA plate. For MCF-7, 8500, 6000, and 3500 cells per well were used for assays corresponding to 24, 48 and 72 h respectively, and for fibroblast cells, 6000, 5000 and 4000 cells per well were used for assays corresponding to 24, 48 and 72 h respectively (number of cells/well for each period of time was calculated based on the standard curves of each cell line). Before beginning the experiment, cells were cultured for 24 h. For analysis of the effects of each compound, in separate reactions, cells were treated with nanoTC, nanoTam, or Tamoxifen at concentrations ranging from 1 to 30 µg/mL or with nanoCur at concentrations ranging from 1 to 60 µg/mL. Diblock polymeric nano-carrier was used at concentrations ranging from 20 to 200 µg/mL as a control reaction to exclude false-positive results due to polymer toxicity. After incubation (24, 48 or 72 h), 20 µL of MTT solution (5 µg/mL) (Sigma-Aldrich, USA) was added to each well, and the plate was incubated for four hours. The supernatant was removed and 20 µL of dimethyl sulfoxide (DMSO) was added to the wells. The absorbance was read at 570 nm. All reactions were repeated three times. 

*Cell Cycle Analysis*

In separate reactions, cells were co-cultured with 1-3 µg/mL below the IC50 of Tamoxifen, nanoTam, nanoCur, and nanoTC. After incubation for 24 h cells were trypsinized and washed twice with PBS. Cells were fixed in 75% Ethanol for 24 h and washed twice with PBS. Cells were stained with 400 µL stain solution (2% of 1 µL/mLPropidium Iodide, 2% of 1 µg/mL RNASA and 0.1% Triton X-100). Cells were then analyzed via FACSCalibur^™ ^flow cytometer (BD Biosciences, USA). DATA were analyzed by Flowing software. 

*Analysis of Apoptosis*

Apoptosis in cells treated with each of the four compounds was analyzed via Annexin-V-Fluos and PI staining kit (Roche, Germany) according to the manufacture’s instruction. FACSCalibur^™ ^flow cytometer (BD Biosciences, USA) and Flowing software were used for analysis of data. 

*Real-Time PCR analysis*

Total RNA was extracted via TRIzol reagent (Life Technologies) from cells treated with each of four compounds for 24 h treatment. Genomic DNA contamination was removed by DNase I (Thermo Fisher Scientific, USA) treatment. RNA was analyzed and quantified by electrophoresis and UV spectrophotometry. cDNA was synthesized using PrimeScript™ RT reagent kit (Takara Bio Inc, Japan). Real-Time PCR was carried out using SYBR Premix Taq^™ ^(Takara, Japan). Results of Real-Time PCR were analyzed using 2-^∆∆Ct ^method. Primers were designed using Oligo, Allel ID and Perl Primer software. Primers.

*Statistical Analysis*

All tests were repeated at least three times and mean was presented as the final result. Student’s *t* test was used to analyze the data. *P* value was used to determine the significance of the results. GraphPad Prism Software v5.0 was used for data analysis.

## Results

*Cell Viability Assay*

**Figure 1:**
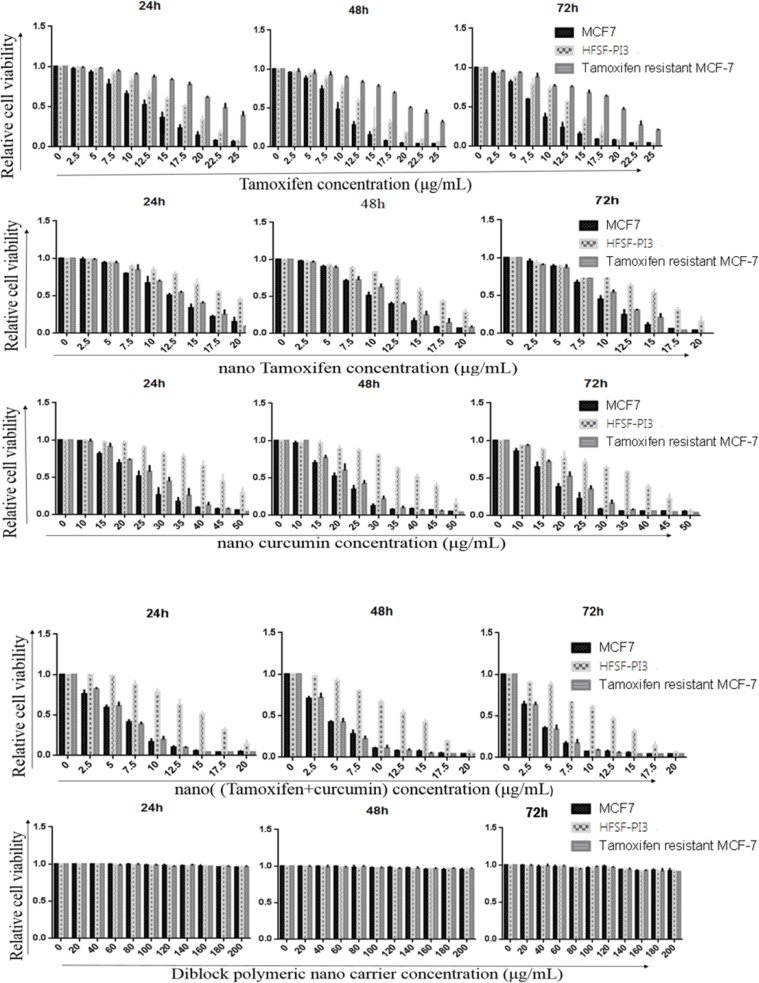
Anti-tumor activity and toxicity of Tamoxifen (A), nano-Tamoxifen (B), nano-Curcumin (C), nano-(Tamixifen+Curcumin) (D) and nano-carrier (E) on TS-MCF-7, TR-MCF-7 and HFSF-PI3 cells on 24, 48 and 72 h. Results show that all four drugs are capable of induction of cell death. In all cases, with the exception of TR-MCF-7 cells treated with Tamoxifen, lower concentration of the drug was needed to induce cell death in cancer whereas normal cells required more concentrated drug for induction of the same effect. Diblocknano-carrier shows no toxicity in normal and cancer cells

MTT assay was used to evaluate and obtain the effective dose of Tamoxifen, nanoTam, nanoCur and nanoTC that can inhibit MCF-7 cancer cells but leave the normal dermal fibroblast (HFSH-PI3) cells unaffected (Figure 1). The *p* value of IC50 difference between MCF-7 and HFSF-PI3 cells treated with Tamoxifen, nanoTam, nanoCur and nanoTC was 0.0041**, 0.0008***, 0.0015 and 0.001, respectively which is considered statistically significant. There was no significant difference in the IC50 between the treatment of Tamoxifen and nanoTam in MCF-7 cells. However, significant difference was seen between the treatment of nanoTC with nanoTam, nanoCur and Tamoxifen (*p* value; 0.0066**, 0.0064** and 0.0007*** respectively). There was no difference in the IC50 of nanoTC, nanoTam and nanoCur between the TS-MCF-7 and TR-MCF7 cells. However, a significant difference was observed in the Tamoxifen treatment of the TS-MCF-7 and TR-MCF-7 cells (*p* value 0.0021**). There was also a significant difference in the Tamoxifen treatment of TR-MCF-7 and HFSF-PI3 cells (*p* value; 0.0068**).

*Analysis of cell cycle in TS-MCF-7 and TR-MCF-7 cells*

**Figure 2:**
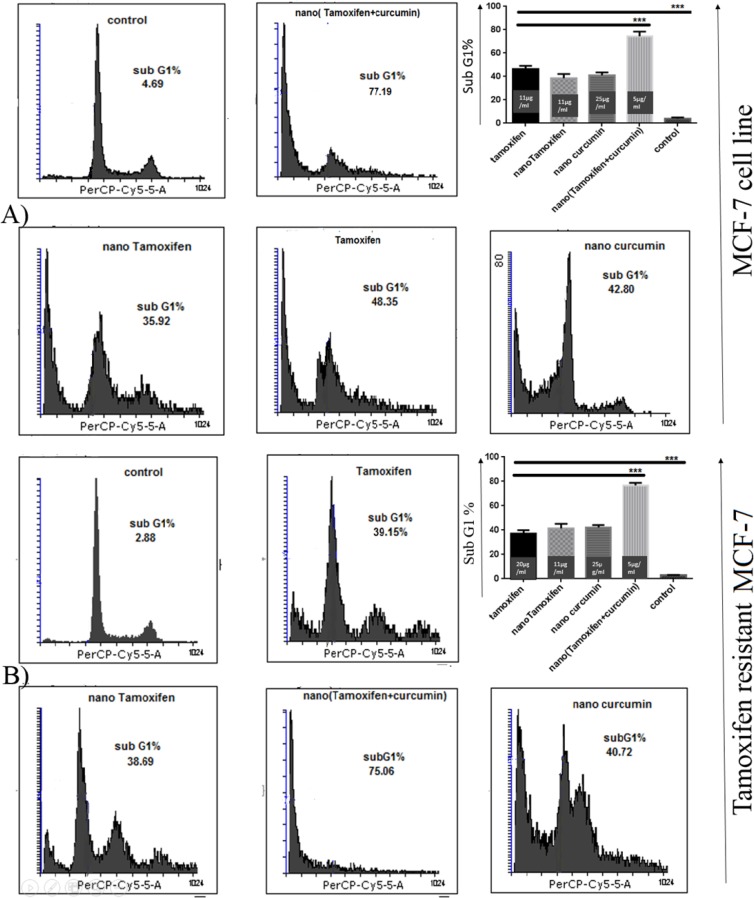
Cell cycle analysis by Flowcytometry. (A); TS-MCF-7, (B); TR-MCF-7. The population of TS-MCF-7 and TR-MCF-7 cells with Sub-G1 DNA content treated with Tamoxifen (11 µg/mL for TS-MCF-7 and 20 µg/mL for TR-MCF-7), nano-Tamoxifen (11 µg/mL), nano-Curcumin (25 µg/mL) and nano-(Tamoxifen+Curcumin) (5 µg/mL). (*P* value < 0.001 ***).

Analysis of cell cycle in MCF-7 cells treated with Tamoxifen, nanoTam, nanoCur and nanoTC in concentrations of 1-3 µg/mL below the IC50 showed that all compounds are able to induce cell death and increase the population of cells with sub-G1 DNA content (Figure 2). 

Nano TC with the formula of 5 µg/mL Tamoxifen and 15 µg/mL Curcumin could induce cell death in 77.19% of TS-MCF-7 cells, whereas same concentrations of Tamoxifen alone or Curcumin alone failed to induce such rate of cell death. The level of cell death induced by the same concentrations of nanoTC on TR-MCF-7 was equal to that of TS-MCF-7 cells, however, for Tamoxifen alone, twice the concentration which was needed for induction of cell death in TS-MCF-7 cells, was required to induce cell death in TR-MCF-7 cells. 

*Analysis of Apoptosis*

**Figure 3:**
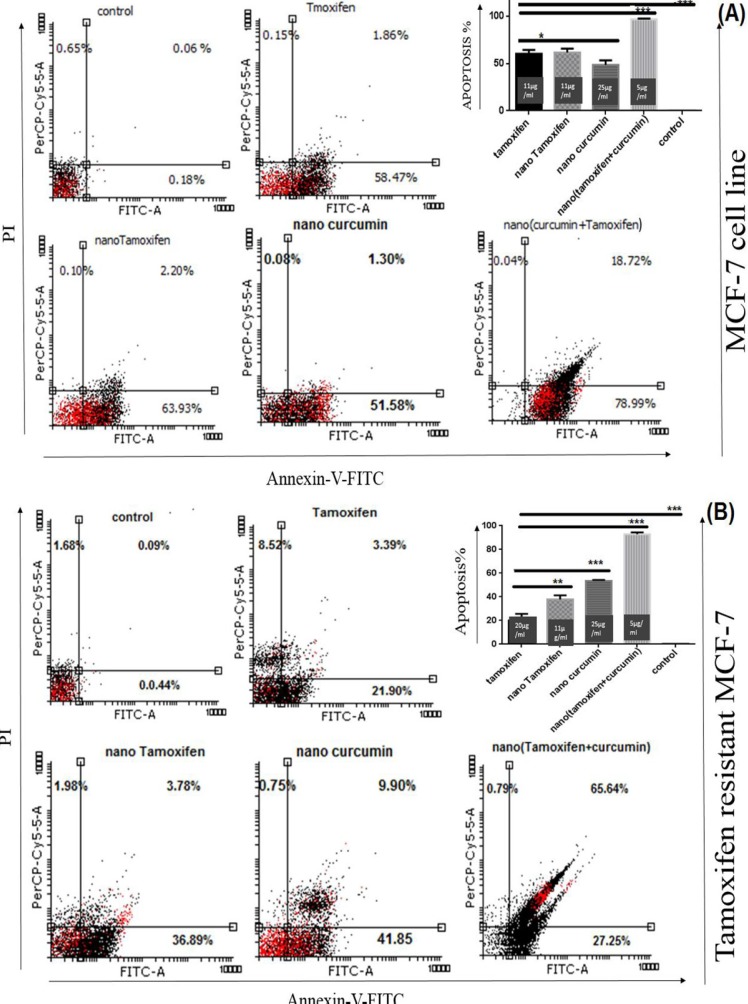
Analysis of apoptosis via Annexin-V-Flus Staining kit. (A); TS-MCF-7, (B); TR-MCF-7. Treatment of TS-MCF-7 and TR-MCF-7 cells with Tamoxifen (11 µg/mL for TS-MCF-7 and 20 µg/mL for TR-MCF-7), nanoTam (11 µg/mL), nanoCur (25 µg/mL) and nanoTC (5 µg/mL). Apoptosis is significantly increased by synergistic effect of Tamoxifen and Curcumin. (*P* value <0.1*), (*P* value <0.01**) and (*P* value <0.001***). The bar graph shows the combination of early and late apoptosis

Annexin-V-Fluos staining kit was used to study cell apoptosis in the treated cells. NanoTC with the formula mentioned above, could induce apoptosis in 98% of TS-MCF-7 cells, while the same concentrations of Tamoxifen alone or Curcumin alone could not produce the same results (Figure 3).

Although twice of the concentration of Tamoxifen needed for induction of cell death in TS-MCF-7 cell line was used for TR-MCF-7 cells, the rate of apoptosis was lower. MTT assay results showed that both TS-MCF-7 cells and TR-MCF-7 cells have the same IC50 for nanoTam. This suggests that the mechanism of Tamoxifen resistance is due to reduced uptake of the drug.

**Figure 4:**
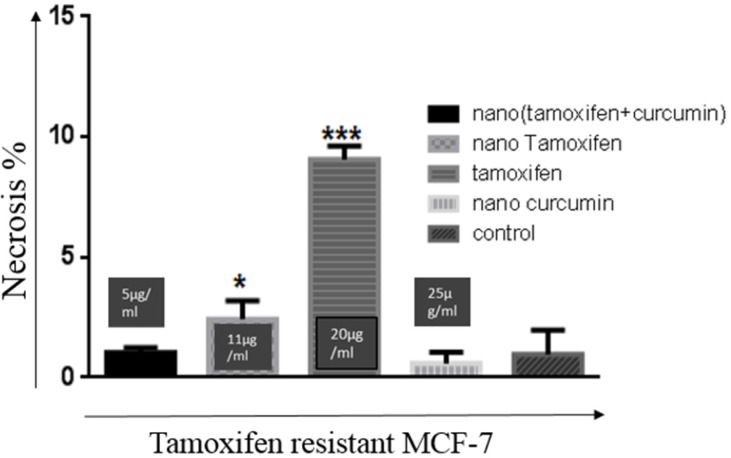
Rate of necrosis in treatment of TR-MCF-7 cells with all four compounds. Concentrations are 20 µg/mL, 11 µg/mL, 25 µg/mL, and 5 µg/mL for Tamoxifen, nanoTam, nanoCur and nanoTC respectively. Compared with other treatments, the rate of necrosis is significantly higher (*P *value< 0.001 ***) in Tamoxifen treatment. Treatment with NanoTam also results in higher rates of necrosis (*P* value< 0.1*) than treatments with nanoCur and nanoTC

An important difference in the nanoTC treatment with the Tamoxifen or nanoTam treatment of TR-MCF-7 cells, is that Tamoxifen or nanoTam treatment usually results in necrosis, which could be the result of toxicity induced by the high dosage of the drug (Figure 4).

Gene expression analysis revealed that Curcumin decreases the expression of anti-apoptotic protein; Bcl-2 and increases the expression of pro-apoptotic protein Bax in cancer cell lines. However, Tamoxifen decreases the expression of Bcl-2 without affecting the expression of Bax protein.

*Real-Time PCR analysis*

**Figure 5:**
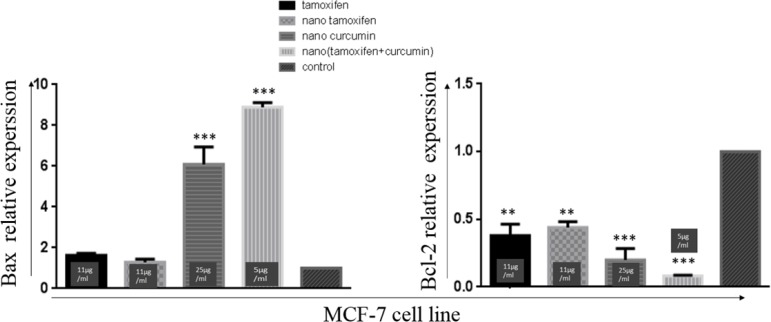
Expression of Bax and BCL-2 in treatment of MCF-7 with Tamoxifen (11 µg/mL), nanoTam (11 µg/mL), nanoCur (25 µg/mL) and nanoTC (5 µg/mL). in this investigation, duplicate aliquots of all RNA samples were used in separate qPCR amplification. *P *value< 0.001*** and *P* value<0.01**.

Tamoxifen and nanoTam treatment significantly reduce the expression of Bcl-2 without affecting the expression of Bax. However, Curcumin can increase the expression of Bax and reduce the expression of Bcl-2 in a statistically significant manner (Figure 5).

NanoTC could increase the Bax expression by 9 folds and decrease the expression of Bcl-2 by -12/5 folds when compared with the control reactions. These changes in the expression of Bax and Bcl-2 were not seen in treatment with Tamoxifen alone or Curcumin alone (at the same concentration used in nanoTC).

## Discussion

In this research the ability of induction of apoptosis and toxicity of modified Tamoxifen was investigated. Tamoxifen was modified in two different approaches; in one strategy, Tamoxifen was packaged with Diblocknano-polymer, and in the second strategy, Tamoxifen was packaged in Diblocknano-polymer with Curcumin. Efficiency and anti-proliferative effect of all compounds was tested on breast cancer cells, both sensitive and resistant to Tamoxifen. Fibroblast cells (HFSF-PI3) were used to assess the toxicity of developed drugs. The packaging of Tamoxifen with nano-polymer increased the IC50 for HFSF-PI3 cells. The higher IC50 of packaged tamoxifen for normal cells allows the drug to be used in higher dosages without affecting the normal cells, thus allowing more effective eradication of cancer. 

In previous studies, various nano-polymers including; Poly (amidoamine) (PAA)-Cholesterol Conjugate Nanoparticles and topical liposomes were used for Tamoxifen delivery. Topical liposomes were used for topical usage of Tamoxifen on the skin and PAA-cholesterol nanoparticles are used for slow release of the drug and improvement of anti-proliferative effects (18, 19).

Formulating Tamoxifen and Curcumin with the Diblocknano-polymer results in a nano-based anti-cancer drug with less toxicity in comparison with Tamoxifen or nanoTam. The drug does not affect or induce apoptosis in normal fibroblast cells at the IC50 concentration of TS-MCF-7 and TR-MCF-7 cells. NanoTC can induce cell death in cancer cells in concentrations much smaller than those needed for Tamoxifen alone or Curcumin alone. This synergistic effect of Curcumin and Tamoxifen allows reducing the Tamoxifen dosage by at least 2 folds while preserving the anti-cancer effect of the drug and furthermore, the new formula overcomes the Tamoxifen resistance developed by long-term usage of the drug. The development of resistance against Tamoxifen is the most significant problem in the treatment of breast cancer. The resistant cells can outgrow the normal population of cells in the tumor and cause a more aggressive form of cancer or relapse, ultimately resulting in Tamoxifen treatment failure. The new formulation of Tamoxifen and Curcumin packaged with Diblocknanopolymer, overcomes this issue and can have the same impact on TR-MCF-7 as TS-MSC-7 cells, causing uniform eradication of both resistant and sensitive cancer cells and more successful treatment. 

Curcumin improves the expression of Bax by P53 mediated pathway, thus increases the population of cells with DNA content of Sub G0/G1 (10). Curcumin activates Caspase 8 which cleaves Bid protein and results in the release of Cytochrome C from the mitochondria and activation of Caspase 9, Caspase 3 and Poly ADP ribosil polymerase (PARP). This cascade will induce apoptosis in cancer cells (20).

On the other hand Tamoxifen reduces the expression of signaling proteins such as protein kinase C (PKC), Calmodulin, Transforming growth factor-beta (TGF beta), and the protooncogen C-myc and induces apoptosis. It was also shown that Caspases and Mitogen-Activated Protein Kinases (MAPK) including C-Jun N-terminal kinase (JNK) and p38 could have a role in the apoptosis induced by Tamoxifen (21).

Flow cytometric analysis of Mithramycin-stained cells revealed that MCF-7 cells treated with Tamoxifen are usually in sub-G1 phase and population of cells in S and G2 phases are significantly decreased (22). 

Analysis of cell cycle and apoptosis showed that nanoTC can induce apoptosis and increase the population of cells with the DNA content of sub-G1 phase in TS-MCF-7 and TR-MCF-7 cells with concentrations much less than nanoTam, Tamoxifen or Curcumin alone. This confirms the synergistic effect of Curcumin and Tamoxifen on induction of apoptosis in cancer cells.

Curcumin restores various resistance mechanisms obtained by Tamoxifen-resistant cells and affects the modified pathways through MAPK, PI3K/Akt, C-myc, Cycline D, NF-kB and SRC to resume sensitivity to Tamoxifen in Tamoxifen-resistant cells (3). 

In previous studies, Curcumin was used alone and in combination with Tamoxifen to overcome the Tamoxifen resistance in cancer cells, which resulted in cell cycle arrest in G2/M phase and induction of apoptosis in those cells. However, nano-carriers were not used for packaging of the drugs. 

The results obtained by our study show more promise and can induce apoptosis in breast cancer cells with much higher efficacy. Although the results of previous studies are statistically significant, lack of proper packaging of a compound such as Curcumin which is lipophilic and biodegradable makes the *in-vivo* application of the drug challenging (3).

Our study showed that TR-MCF-7 cells require more dosage of Tamoxifen for induction of cell death and increase the population of cells with DNA content of sub-G1 phase. Analysis of cells treated with Tamoxifen revealed that in such high dosages, a portion of cell death is due to necrosis. Further studies showed that nanoTam, nanoCur, and nanoTC induce cell death in both TS-MCF-7 and TR-MCF-7 cells at the same concentration. Although, necrosis is also seen in the treatment by nanoTam, the percentageis in much smaller scale than the treatment with Tamoxifen alone. Overall, results indicate that formulating Tamoxifen with Curcumin and proper packaging of the drug can significantly increase the efficacy of the drugs and overcome the major drawback of Tamoxifen which is a development of Tamoxifen resistant cells. 

## Conclusion

Modification of Tamoxifen by packaging it with Diblocknano-polymer or formulating the drug with Curcumin can significantly increase the efficacy of the drug in the treatment of breast cancer. Packaging of the drug with the nano-polymer can probably facilitate the entry of Tamoxifen and overcome the resistance-mechanisms obtained by drug-resistant cancer cells and furthermore, reduce the toxicity of the drug on cells.
